# Resting-state functional connectivity for determining outcomes in upper extremity function after stroke: A functional near-infrared spectroscopy study

**DOI:** 10.3389/fneur.2022.965856

**Published:** 2022-11-09

**Authors:** Youxin Sui, Chaojie Kan, Shizhe Zhu, Tianjiao Zhang, Jin Wang, Sheng Xu, Ren Zhuang, Ying Shen, Tong Wang, Chuan Guo

**Affiliations:** ^1^Department of Rehabilitation Medicine, The First Affiliated Hospital of Nanjing Medical University, Nanjing, China; ^2^School of Rehabilitation Medicine, Nanjing Medical University, Nanjing, China; ^3^Department of Rehabilitation Medicine, Changzhou Dean Hospital, Changzhou, China

**Keywords:** stroke, functional near-infrared spectroscopy (fNIRS), functional connectivity, resting-state, Fugl-Meyer, upper extremity

## Abstract

**Objective:**

Functional near-infrared spectroscopy (fNIRS) is a non-invasive and promising tool to map the brain functional networks in stroke recovery. Our study mainly aimed to use fNIRS to detect the different patterns of resting-state functional connectivity (RSFC) in subacute stroke patients with different degrees of upper extremity motor impairment defined by Fugl-Meyer motor assessment of upper extremity (FMA-UE). The second aim was to investigate the association between FMA-UE scores and fNIRS-RSFC among different regions of interest (ROIs) in stroke patients.

**Methods:**

Forty-nine subacute (2 weeks−6 months) stroke patients with subcortical lesions were enrolled and were classified into three groups based on FMA-UE scores: mild impairment (*n* = 17), moderate impairment (*n* = 13), and severe impairment (*n* = 19). All patients received FMA-UE assessment and 10-min resting-state fNIRS monitoring. The fNIRS signals were recorded over seven ROIs: bilateral dorsolateral prefrontal cortex (DLPFC), middle prefrontal cortex (MPFC), bilateral primary motor cortex (M1), and bilateral primary somatosensory cortex (S1). Functional connectivity (FC) was calculated by correlation coefficients between each channel and each ROI pair. To reveal the comprehensive differences in FC among three groups, we compared FC on the group level and ROI level. In addition, to determine the associations between FMA-UE scores and RSFC among different ROIs, Spearman's correlation analyses were performed with a significance threshold of *p* < 0.05. For easy comparison, we defined the left hemisphere as the ipsilesional hemisphere and flipped the lesional right hemisphere in MATLAB R2013b.

**Results:**

For the group-level comparison, the one-way ANOVA and *post-hoc* t-tests (mild vs. moderate; mild vs. severe; moderate vs. severe) showed that there was a significant difference among three groups (*F* = 3.42, *p* = 0.04) and the group-averaged FC in the mild group (0.64 ± 0.14) was significantly higher than that in the severe group (0.53 ± 0.14, *p* = 0.013). However, there were no significant differences between the mild and moderate group (MD ± SE = 0.05 ± 0.05, *p* = 0.35) and between the moderate and severe group (MD ± SE = 0.07 ± 0.05, *p* = 0.16). For the ROI-level comparison, the severe group had significantly lower FC of ipsilesional DLPFC–ipsilesional M1 [*p* = 0.015, false discovery rate (FDR)-corrected] and ipsilesional DLPFC–contralesional M1 (*p* = 0.035, FDR-corrected) than those in the mild group. Moreover, the result of Spearman's correlation analyses showed that there were significant correlations between FMA-UE scores and FC of the ipsilesional DLPFC–ipsilesional M1 (*r* = 0.430, *p* = 0.002), ipsilesional DLPFC–contralesional M1 (*r* = 0.388, *p* = 0.006), ipsilesional DLPFC–MPFC (*r* = 0.365, *p* = 0.01), and ipsilesional DLPFC–contralesional DLPFC (*r* = 0.330, *p* = 0.021).

**Conclusion:**

Our findings indicate that different degrees of post-stroke upper extremity impairment reflect different RSFC patterns, mainly in the connection between DLPFC and bilateral M1. The association between FMA-UE scores and the FC of ipsilesional DLPFC-associated ROIs suggests that the ipsilesional DLPFC may play an important role in motor-related plasticity. These findings can help us better understand the neurophysiological mechanisms of upper extremity motor impairment and recovery in subacute stroke patients from different perspectives. Furthermore, it sheds light on the ipsilesional DLPFC–bilateral M1 as a possible neuromodulation target.

## Introduction

Stroke is a neurological disorder caused by vascular dysfunction in the brain and is a leading cause of disability (1). The impairment of upper limb motor function is common after stroke and may seriously impact patients' quality of life (2). It makes sense that loss of upper extremity function would be caused by damage to brain regions. However, accumulating evidence has demonstrated that focal lesions caused by stroke can affect not only perilesional brain regions but also distal brain regions, which may cause adjustable reorganization of neural networks (3, 4).

Resting-state functional connectivity (RSFC) is a powerful method for mapping functional networks in the brain, defined as the temporal correlation of the blood oxygenation-level-dependent (BOLD) signal across regions without any imposed task (5, 6). According to the mechanism of “neurovascular coupling,” corresponding hemodynamic responses can be induced by neural activity due to the increased oxygen demand in activated brain areas (7, 8). Therefore, monitoring the hemodynamic fluctuations in brain tissue can provide insight into how activity is organized. When neurons are at rest, their oxygen extraction percentage remains relatively unchanged, resulting in a constant ratio of oxygenated to deoxygenated blood in the surrounding capillary bed.

Resting-state brain networks during stroke recovery were initially assessed using functional magnetic resonance imaging (fMRI). According to studies on resting-state fMRI, stroke patients have different modes of functional connectivity compared with healthy people, and abnormal dynamic functional connectivity is associated with post-stroke motor recovery (4, 9–11). Although fMRI is the gold standard for measuring cortical activity, it has some drawbacks, such as restricted monitoring environments, acoustic scanner noises, subject head immobilization, and high cost (12). It might not be appropriate for people with metal implants, claustrophobia, or hyperactivity (13).

Functional near-infrared spectroscopy (fNIRS) is a non-invasive neuroimaging tool which can constantly monitor regional tissue oxygenation by recording the concentrations of both oxygenated hemoglobin (HbO) and deoxygenated hemoglobin (HbR) (14). The fNIRS is more portable and less noisy than fMRI, so it is more acceptable for patients during resting mode (15, 16). A concurrent recording study found that fNIRS-RSFC values correlated directly with fMRI-RSFC values, indicating that optical brain connectivity is associated with functional brain architecture (17). Moreover, Zhang et al. conducted a test–retest analysis to identify the reliability fNIRS-RSFC. They found that individual-level RSFC shows good to excellent map-/cluster-wise reliability for HbO signals, and group-level RSFC shows exceptional reliability (18). These prove that fNIRS-RSFC has good reliability and validity. Given that fNIRS mainly monitors hemodynamic changes in the superficial layer of the brain tissue (19), we believe that fNIRS could be an appropriate monitoring tool to investigate the cerebral cortical alterations related to upper limb motor recovery (20).

Studies using imaging technologies, such as fMRI and fNIRS, have shown that stroke patients with upper extremity motor deficits have different RSFC patterns from healthy people (4, 21, 22). However, the stroke patients enrolled in these studies varied greatly in terms of the time post-stroke and the severity of motor impairments. Our study focused on the post-stroke motor recovery and cortical changes in subacute phase, when the primary lesion is relatively stable (23) and functional impairment becomes a major concern (24). The primary objective of our study was to investigate the different patterns of fNIRS-RSFC among subacute stroke patients with different degrees of upper limb motor dysfunction determined by Fugl-Meyer assessment of upper extremity motor scale (FMA-UE), a reliable method for measuring post-stroke upper extremity motor function (2). We further aimed to assess the correlations between FMA-UE scores and fNIRS-RSFC among the different brain areas.

## Methods

### Participants

This is a cross-sectional observational study. Stroke patients with upper extremity hemiplegia were recruited from the Rehabilitation Medicine Center in Changzhou Dean Hospital between June 2021 and January 2022. The inclusion criteria were as follows: a first-ever unilateral stroke with upper extremity motor deficit; subcortical lesion; post-stroke time between 2 weeks and 6 months; and 30–80 years old. The exclusion criteria were as follows: presence of any other neurological disorder or psychiatric disease; severe cognitive impairment or aphasia; skull defects; and inability to remain seated for 10 min quietly.

All stroke patients who meet the entry requirements will undergo clinical information collection, FMA-UE assessment, and a 10-min fNIRS resting-state monitoring by a blind and trained investigator. The FMA-UE is a validated upper extremity motor impairment scale with excellent inter- and intra-rater reliability (2, 25). It has four subsections, namely, shoulder arm, wrist, hand, and coordination and speed, and was made up of 33 items that are assessed on an ordinal scale of 0 (absence), 1 (partial impairment), and 2 (no impairment), giving possible values ranging from 0 to 66. We divided all participants into three groups with different degrees of upper limb motor dysfunction based on FMA-UE scores (26). They are mild impairment group (FMA-UE: 43–66), moderate impairment group (FMA-UE: 29–42), and severe impairment group (FMA-UE: 0–28).

The experimental procedure was approved by the Human Ethics Committee of Changzhou Dean Hospital (CZDALL-2021-001) and was registered in the China Clinical Trial Registration Center (ChiCTR2100047442).

### Data acquisition

**Figure 1:**
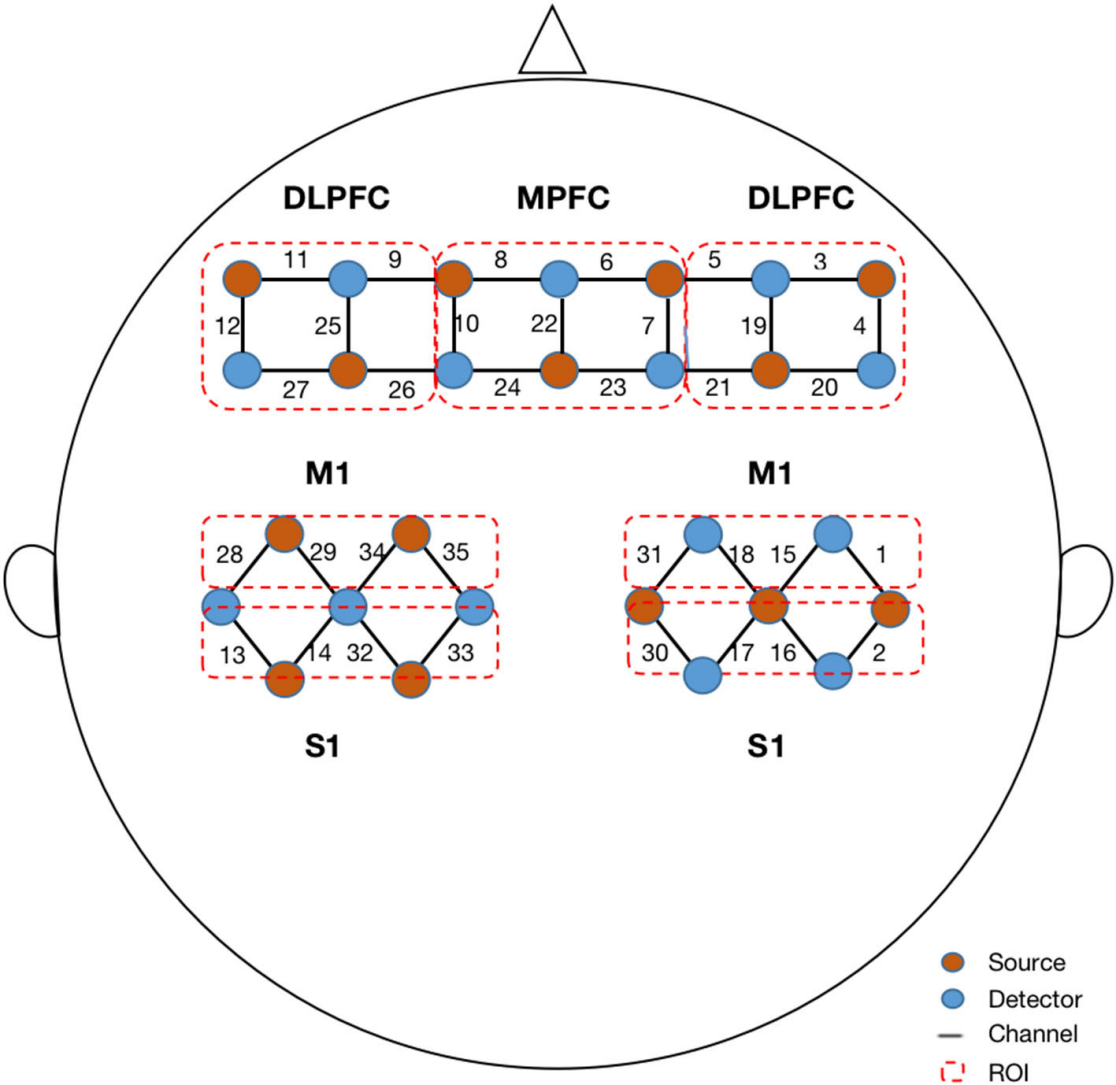
Configuration of fNIRS channels. The red dots represent the light sources, and the blue dots represent the light detectors. In total, 14 sources and 14 detectors resulted in 35 channels encompassing seven regions of interest, specifically bilateral dorsal lateral prefrontal cortex (DLPFC), middle prefrontal cortex (MPFC), bilateral primary motor cortex (M1), and bilateral primary sensory cortex (S1).

The fNIRS signals were acquired using a multichannel fNIRS system (NirScan-6000C, Danyang Huichuang Medical Equipment Co., Ltd., China) with three wavelengths (730, 808, and 850 nm) at a sampling rate of 11 Hz. Based on the 10/20 system, 14 sources and 14 detectors were placed on the bilateral dorsolateral prefrontal cortex (DLPFC), middle prefrontal cortex (MPFC), bilateral primary motor cortex (M1), and primary somatosensory cortex (S1), totaling 35 channels (Figure 1). According to the standard brain localization, the specific correspondence between the channels and the Brodmann brain region overlap is shown in Supplementary material.

During the 10-min fNIRS resting-state monitoring, participants were instructed to remain still and close their eyes without falling asleep. Such resting-state recordings do not require additional perceptual input or behavioral output. The output parameters were the optical densities of the three wavelengths, which were then converted to concentration changes in oxyhemoglobin (HbO) and deoxyhemoglobin (HbR) based on the modified Beer–Lambert law. The detectors and light sources were secured using a flexible headgear to ensure that they were in direct contact with the skin as much as possible to obtain high-precision data. The average distance between the light detectors and sources was set to 30 mm.

### Pre-processing

From the continuous time course, we extracted 5 min of data for each subject (ranging from 3 to 8 min). Because the sampling rate was 11 Hz, the maximum number of time points obtained from each channel for one person was 3,300 (5 × 60 × 11). Due to uncontrollable experimental factors, such as unconscious coughs or yawns, motion artifacts were inevitable. We used visual inspection and calculated the coefficient of variation (CV) of each raw data, a common process for multichannel NIRS measurements, to control the data quality:


$$\begin{array}{l} CV=\frac{\sigma }{\mu }\times 100\%. \end{array}$$

Here, σ is the temporal standard deviation for a data channel and μ is the corresponding mean value. Measurement data with a CV exceeding 15% were rejected (27, 28).

Raw data were preprocessed using the HOMER2 toolbox (version 2.8), which is a built-in toolbox of MATLAB R2013b (MathWorks, Natick, MA, USA). The pre-processing procedures were as follows: (1) the raw NIRS light intensity was converted to an optical density signal; (2) the HOMER2 built-in function was used to detect motion artifacts by channel (parameters set as tMotion = 1 s; tMAsk = 2.0; STDEVthresh = 15.0; AMPthresh = 5.0); (3) correct motion artifacts were detected by the spline interpolation method (hmrMotionCorrectSpline); (4) filtration, the majority of the systemic hemodynamic components were removed with a band-pass (0.01–0.1 Hz) filter, such as those originating from cardiac cycles (~1 Hz) and respiration (~0.2–0.3 Hz) (29); and (5) the filtered optical density data were converted into oxy-Hb and deoxy-Hb by applying the modified Beer–Lambert law (30). A differential path length factor (DPF) of 6 was set for this study, accounting for the true effective path length between the source and detector (31). HbO signals were chosen for further analysis because they have been shown to have larger effects in fNIRS connectivity analysis and are more sensitive to cerebral vascular alterations than HbR signals (32, 33).

For easy comparison, brains of 20 right hemisphere affected patients (3 from the mild group, 6 from the moderate group, and 11 from the severe group) were flipped, defining the left hemisphere as the ipsilesional hemisphere and the right hemisphere as the contralesional hemisphere.

### Functional connectivity

For each participant, Functional Connectivity (FC) was calculated by correlation coefficients with the 5-min filtered signals between the 35 measurement channels, which describes the linear correlation between the two time-domain signals with values ranging from −1 to 1 (34). The formula used is as follows:


$$\begin{array}{l} r=\frac{\sum_{i=1}^{n}\left(X_{i}-\bar{X}\right)\left(Y_{i}-\bar{Y}\right)}{\sqrt{{\sum_{i=1}^{n}\left(X_{i}-\bar{X}\right)}^{2}}\sqrt{{\sum_{i=1}^{n}\left(Y_{i}-\bar{Y}\right)}^{2}}} \end{array}$$

where X and Y are the time series of hemoglobin concentrations in the various channels and r is the correlation coefficient. The procedure generated a 35 × 35 correlation matrix for each participant. Then, we divided all channels into seven ROIs (ipsilesional DLPFC, MPFC, contralesional DLPFC, ipsilesional M1, contralesional M1, ipsilesional S1, and contralesional S1) and calculated the ROI-to-ROI correlation coefficients for each patient (Figure 1). This procedure generated a 7 × 7 correlation matrix for each participant. In addition, we averaged the time series of all channel pairs for each patient, which contributed to the general FC value for each patient. Therefore, for each participant, a 35 × 35 correlation matrix was calculated between each pair of channels, a 7 × 7 correlation matrix was calculated between each pair of ROIs, and a general FC value was calculated for group comparison.

### Statistical analysis

Statistical analyses were performed using MATLAB R2013b and IBM SPSS Statistics 25 (IBM Inc., New York, USA). For comparison of demographic characteristics, one-way analysis of variance (ANOVA) was adopted for age, Mini-Mental State Examination, and time post-stroke among the three patient groups. To reveal the comprehensive differences in FC among the three groups of patients with different degrees of upper limb motor dysfunction, we compared FC at the group level and the ROI level. For group-level comparison, the correlation matrices of all participants in one group were averaged, one-way ANOVA was used to compare the average connectivity among groups, and post-hoc t-tests between the individual groups (mild vs. moderate; mild vs. severe; moderate vs. severe) were archived by applying the Bonferroni test. For ROI-level comparison within groups, the correlation matrices of all participants in one group were shown as a 7 × 7 × n (number of patients in each group) matrix, and the t-test was used for ROI pair-wise comparisons. For multiple comparisons, the false discovery rate (FDR) correction was used. We defined a significant difference in this study as both FDR-corrected q < 0.05 and power >0.8. We then performed a linear regression analysis to detect possible factors influencing FC. Additionally, to determine the associations between the FC of different ROI–ROI pairs and FMA-UE scores, a non-parametric Spearman correlation was performed according to the result of the Shapiro–Wilk normality test. A *p* < 0.05 was considered to be statistically significant.

## Results

### Demographics

Data were obtained from 74 stroke patients. After visual inspection and CV calculation, the data from 25 patients (5 from the mild, 5 from the moderate, and 15 from the severe group) were excluded. Finally, the data from the remaining 49 participants were analyzed, including 17 in the mild, 13 in the moderate, and 19 in the severe group. The one-way ANOVA results for the demographics of the three groups are listed in Table 1. The three groups showed no significant differences in age, time post-stroke, and Mini-Mental State Examination scores.

**Table 1:** Demographic and clinical data of stroke patients.

	**Mild group (*n* = 17)**	**Moderate group (*n* = 13)**	**Severe group (*n* = 19)**	***F***	***p***
Age(years)	65.47 ± 11.94	65.92 ± 10.69	65.42 ± 9.33	0.01	0.99
Time post-stroke (days)	43.12 ± 36.12	51.77 ± 45.08	58.89 ± 50.50	0.56	0.57
MMSE	25.00 ± 7.84	25.54 ± 7.08	20.05 ± 7.83	2.69	0.08
FMA-UE	52.35 ± 6.39	35.38 ± 3.66	11.63 ± 9.44	–	–
Sex (male/female)	14/3	9/4	14/5	–	–
Stroke type (I/H)	14/3	12/1	14/5	–	–
Damaged hemisphere (left/right)	14/3	7/6	8/11	–	–

MMSE, Mini-Mental State Examination; FMA-UE, Fugl-Meyer Motor Upper Extremity Scale; I, Ischemic; H, Hemorrhagic.

### Group-based functional connectivity

**Figure 2:**
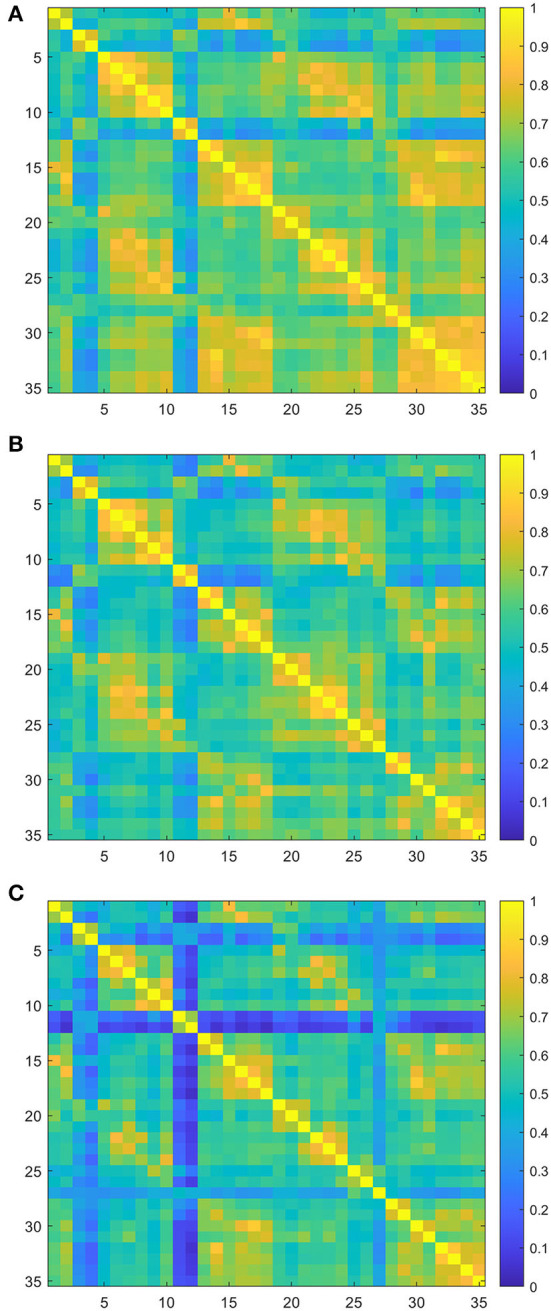
Group-averaged resting-state functional connectivity (RSFC) matrix diagram. Axes represent the channels. Each channel with its correlation coefficient set at zero (the diagonal line). **(A)** RSFC matrix of the mild group. **(B)** RSFC matrix of the moderate group. **(C)** RSFC matrix of the severe group.

The group-averaged FC for each group is shown in Figure 2. There was a significant difference in FC values among three groups, and the higher the degree of dysfunction, the lower the averaged connectivity strength. Quantitatively, the mean values of connectivity strength and its standard deviations were 0.64 ± 0.14 for the mild group, 0.59 ± 0.11 for the moderate group, and 0.53 ± 0.14 for the severe group (Figure 2). One-way ANOVA of the group-averaged functional connectivity of HbO signals showed a significant difference among three groups (*F* = 3.42, *p* = 0.04). The Bonferroni *post-hoc* test revealed that the group-averaged FC in the mild group was significantly higher than that in the severe group (MD ± SE = 0.12 ± 0.04, *p* = 0.013). However, there were no significant differences between the mild and moderate group (MD ± SE = 0.05 ± 0.05, *p* = 0.35) and between the moderate and severe group (MD ± SE = 0.07 ± 0.05, *p* = 0.16).

### ROI-based functional connectivity

**Figure 3:**
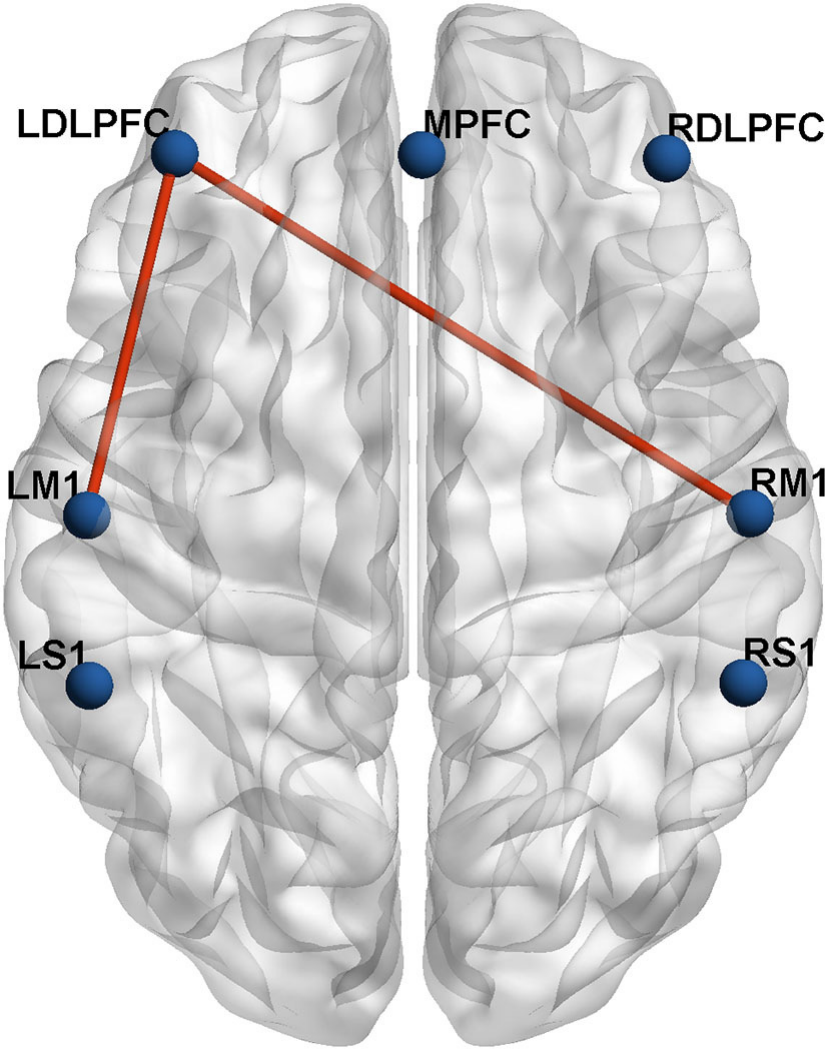
The inter-group differences in actual ROIs represented by automated anatomical labeling (AAL) atlas in axial view. The blue nodes represent the seven regions of interest. The red lines represent the connections with significant differences (*p* < 0.05) between the mild group and severe group.

To investigate the characteristics of the ROI–ROI connection, the internal channels of the seven ROIs were averaged, and two-sample t-tests with FDR correction were used to examine the differences between the two groups. In the lesioned hemisphere, there was a significant change in long-distance connectivity associated with the ipsilesional DLPFC. The severe group had much lower brain connection intensity between the ipsilesional DLPFC and ipsilesional M1 (mild group: 0.77 ± 0.12, severe group: 0.55 ± 0.22, *p* = 0.015, FDR-corrected), as well as between the ipsilesional DLPFC and contralesional M1 (mild group: 0.70 ± 0.22, severe group: 0.45 ± 0.24, *p* = 0.035, FDR-corrected) than the mild group (Figure 3). In addition, the inter-hemispheric connection between the ipsilesional DLPFC and ipsilesional M1 is stronger than the intra-hemispheric connection between the ipsilesional DLPFC and contralesional M1 in both mild and severe groups.

### Relationship between FC and FMA-UE

**Figure 4:**
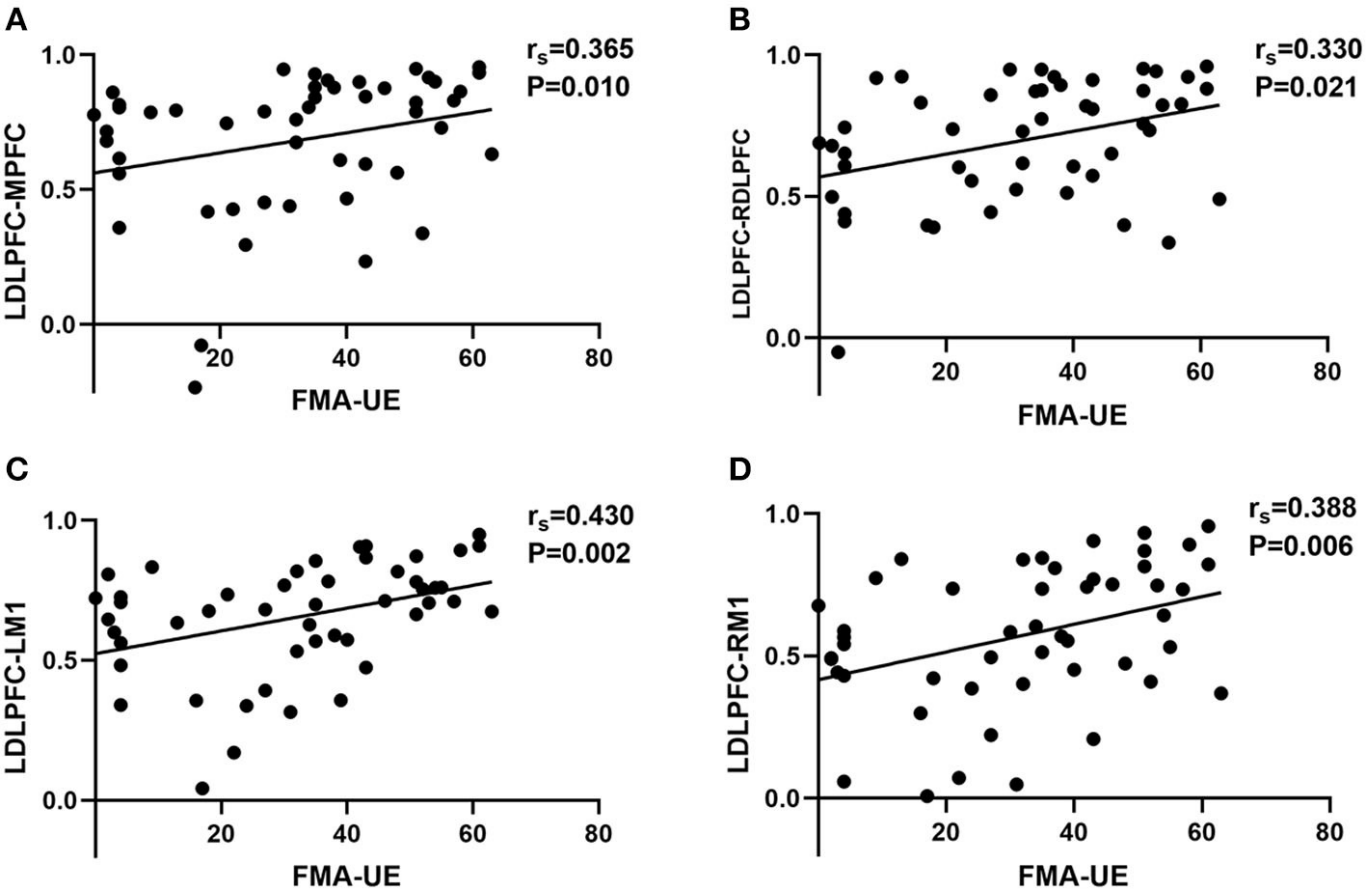
The correlation scatter diagrams of Spearman's correlation analysis between FMA-UE scores and RSFC among different ROIs. LDLPFC, left dorsolateral prefrontal cortex; MPFC, middle prefrontal cortex; RDLPFC, right dorsolateral prefrontal cortex; LM1, left primary motor cortex; RM1, right primary motor cortex. We defined left hemisphere is ipsilesional hemisphere and right hemisphere is contralesional hemisphere. **(A)** The significant correlation between FMA-UE and FC of LDLPFC–MPFC. **(B)** The significant correlation between FMA-UE and FC of LDLPFC–RDLPFC. **(C)** The significant correlation between FMA-UE and FC of LDLPFC–LM1. **(D)** The significant correlation between FMA-UE and FC of LDLPFC–RM1. The “rs” is the correlation coefficient of Spearman's analysis.

The multiple linear regression results showed that there was a positive effect of FMA-UE on FC, but no significant effect of age, sex, or time post-stroke. Spearman's correlation analyses showed the relationship between the FC of 21 ROI pairs (e.g., ROI1–ROI2 and ROI1–ROI3) and FMA-UE scores. FC of four ROI pairs was found to be positively associated with FMA-UE (Figure 4): ipsilesional DLPFC–ipsilesional M1 (*r* = 0.430, *p* = 0.002), ipsilesional DLPFC–contralesional M1 (*r* = 0.388, *p* = 0.006), ipsilesional DLPFC–MPFC (*r* = 0.365, *p* = 0.01), and ipsilesional DLPFC–contralesional DLPFC (*r* = 0.330, *p* = 0.021).

## Discussion

The aim of this study was to explore the patterns of fNIRS-RSFC in stroke patients with different FMA-UE outcomes. In addition, we investigated the relationship between RSFC of ROI pairs and FMA-UE scores. We found that the features of FC were considerably different among the three groups with different degrees of upper extremity motor dysfunction and were correlated with FMA-UE scores.

From the results of group-level comparison, groups with different degrees of upper limb dysfunction showed significant differences in cortical connectivity. Additionally, as the level of impairment increased, the network balance was more disrupted. This finding implies a relationship between the degree of upper limb motor impairment and post-stroke cortical connectivity patterns which is consistent with the fMRI studies. Bonkhoff et al. investigated the difference in resting-state fMRI-based dynamic FC in three stroke severity groups (mildly, moderately, and severely affected) defined by the National Institutes of Health Stroke Scale score (NIHSS). Significant dynamic pattern differences were found in three groups, and they discovered that severe stroke patients had more damaged dynamic connectivity (35). In another fMRI study, 24 stroke patients were divided into two subgroups: completely paralyzed hands (CPH) and partially paralyzed hands (PPH), according to FMA-UE scores. This study showed different patterns of FC in two groups, and the CPH group exhibited significantly reduced FC compared with the PPH group, mainly in the motor-related brain areas (21). Combined with the results of our research, we believe that stroke patients with varied degrees of motor impairment exhibit different patterns of cortical connectivity and cortical connections are usually more severely damaged with heavier impairment.

Further, we compared the differences of FC among different ROIs in three groups. We found that compared with the severe group, the mild group had strengthened connectivity in the ipsilesional DLPFC–ipsilesional M1 and ipsilesional DLPFC–contralesional M1. This result suggested that connectivity between the M1 and DLPFC may be a potential mechanism underlying motor function recovery. The M1 and DLPFC are the main components of the frontal lobe, playing important roles in human behavior, especially in making difficult decisions, and interactions (36). Dysfunction in the DLPFC can negatively impact higher-level cognitive processes (37), particularly executive function (38, 39), and impairment of M1 can result in weakness and impaired motor execution (36). Lefebvre et al. used fMRI to explore the neural mechanisms of motor skill learning in chronic stroke patients. They recruited 23 stroke patients to perform a visuomotor skill with the hemiplegic upper limb and discovered that this motor learning significantly activated the DLPFC and the dorsal premotor cortex of the lesioned hemisphere (40). Gyulai et al. applied EEG to detect the neural activity of finger tapping in 15 mild upper limb paretic stroke patients. The result showed that the DLPFC has cognitive control over fine motor skills, which is linked to post-stroke motor recovery of the lesioned hemisphere (41). These results support the connection between the ipsilesional DLPFC and motor cortex discovered in our study, indicating that the DLPFC and motor cortex are closely associated during motor recovery in stroke patients. On this basis, we found that the inter-hemispheric connection of ipsilesional DLPFC–ipsilesional M1 is stronger than intra-hemispheric connection of ipsilesional DLPFC–contralesional M1 in both mild and severe groups. The neuronal reorganization may occur in both the ipsilesional and contralesional hemispheres to regain motor functionality (42). Reorganization of the ipsilateral hemisphere has traditionally been believed to be more important for motor recovery, and our study confirms this to some extent.

According to the findings of our correlation analysis, we further confirmed the relationship between cortical connection and post-stroke upper extremity dysfunction. The association between FC of ipsilesional DLPFC–bilateral M1 is tightly connected. We assumed that post-stroke recovery of upper limb motor function may be correspondence with the connection between DLPFC and M1. As found in a fMRI study, hand motor recovery after cortical sensorimotor stroke dynamics was linearly correlated with gray matter volumetric increase in the ipsilesional DLPFC using fMRI (43). Oveisgharan applied transcranial direct current stimulation (tDCS) on acute ischemic stroke patients. He found that stimulation of the left DLPFC in conjunction with M1 stimulation of the affected hemisphere led to better upper extremity motor recovery than M1 stimulation alone (44). Another tDCS study also showed that unihemispheric concurrent dual-site anode tDCS of the M1-DLPFC increased motor-evoked potentials by 50% and the effects extended for at least 24 h (45). Combined with the result in our study, we speculate that enhanced cortical connectivity between DLPFC and M1 may promote recovery of motor function after stroke. Additional significant FC related to FMA-UE was found in ipsilesional DLPFC–MPFC and ipsilesional DLPFC–contralesional DLPFC. The MPFC here is mainly about the area of BA10, which occupies the most rostral portions of the superior frontal gyrus and the middle frontal gyrus (46). Both MPFC and DLPFC are important components of the prefrontal lobe that plays an important role in motor control and generation (47). Previous studies have shown persistent and ramping neural activity in frontal cortex predicts specific movements (48). Our result further confirmed the relationship between motor recovery and cortical change in prefrontal cortex.

Growing evidence suggests that stroke is a large-scale network dysfunction that extends beyond the lesioned region (3, 4). One advantage of fNIRS is that it can be mapped to different brain regions depending on the nodes location, capturing the hemodynamic changes in the corresponding brain cortex. It has been demonstrated that fNIRS-derived RSFC is a trustworthy biomarker when interpreted in map- and cluster-wise manners (18, 49). Therefore, it might be meaningful to pay attention to the cortical reconfiguration that results from stroke motor recovery (50).

There are some limitations in our study. First, the referenced classification of upper limb impairment was based on the cluster analysis results of FMA-UE in patients with chronic stroke. The stroke patients we recruited were primarily in the subacute stage. Although the time post-stroke may not be consistent, the FMA-UE scores and FC of each patient were recorded at the same time point, which may reflect the relationship between cortex reorganization and behavior to some extent. Second, the total sample size in our study was not large enough, resulting in unequal numbers in each group. And as this was a cross-sectional observational study, the lesion site and affected side were not strictly limited. We emulated the method in fNIRS (51) and fMRI research (52) to flip the lesioned hemisphere. In future, we hope to carry out fNIRS studies with a larger sample size and prescribed the damaged hemisphere. In addition, fNIRS has limitations as an optical imaging technique, including insufficient anatomical specificity, suboptimal temporal resolution, and low inter-subject reproducibility for individual analysis. Because the spatial resolution of fNIRS is limited by the number of sources and detectors (optodes), its spatial resolution is lower than that of fMRI (53). However, it can be used as a complementary method to predict the extent of recovery from stroke motor deficits.

## Conclusion

This study described different patterns of RSFC among subacute stroke patients with different degrees of upper extremity motor dysfunction. Our results revealed a unique connection between the ipsilesional DLPFC and bilateral M1 during upper extremity motor recovery. The association between FMA-UE scores and RSFC of ipsilesional DLPFC-associated ROI pairs supported the fact that the ipsilesional DLPFC may play a key role in motor-related plasticity. These findings could contribute to a better understanding of the neurophysiological mechanisms of motor impairment and recovery in patients with subacute stroke from various perspectives. It also provides a different perspective of serving the ipsilesional DLPFC-M1 as a stimulating target for neuromodulation.

## Data availability statement

The original contributions presented in the study are included in the article/Supplementary material, further inquiries can be directed to the corresponding authors.

## Ethics statement

The studies involving human participants were reviewed and approved by Human Ethics Committee of Changzhou Dean Hospital. The patients/participants provided their written informed consent to participate in this study.

## Author contributions

YSu, SZ, CK, and CG were in charge of the experimental design. SZ and YSu take charge of the data analysis. CG, YSh, and TW made final decisions during the whole process. All authors participate in the implement of the experiment and the writing of this article. All authors contributed to the article and approved the submitted version.

## Funding

This study was funded by the National Key R&D Program of China (Grant Nos. 2018YFC2001600 and 2018YFC2001603), the Nanjing Municipal Science and Technology Bureau (Grant No. 2019060002), and the Changzhou Municipal Health Commission (Grant No. QN202134).

## Conflict of interest

The authors declare that the research was conducted in the absence of any commercial or financial relationships that could be construed as a potential conflict of interest.

## Publisher's note

All claims expressed in this article are solely those of the authors and do not necessarily represent those of their affiliated organizations, or those of the publisher, the editors and the reviewers. Any product that may be evaluated in this article, or claim that may be made by its manufacturer, is not guaranteed or endorsed by the publisher.
