# Supplementary material for: Resting-state functional connectivity for determining outcomes in upper extremity function after stroke: A functional near-infrared spectroscopy study
Source: Front Neurol. 2022 Nov 9;13:965856. doi: 10.3389/fneur.2022.965856 (PMC9682186; doi:10.3389/fneur.2022.965856)
Supplement: Supplementary file 1 [file Table_1.DOCX]

**The MNI coordinates and anatomical labels corresponding to the measurement channels.**

| **Channel number** | **S-D** | **X/Y/Z** | **Brodmann area overlap** | **Proportion** | **ROI** |
| --- | --- | --- | --- | --- | --- |
| CH01 | S1-D1 | 64/−16/46 | 6 - Pre-Motor and Supplementary Motor Cortex | 88.48% | M1 |
|  |  |  | 4 - Primary Motor Cortex | 8.64% |  |
| CH02 | S1-D6 | 64/−39/49 | 1 - Primary Somatosensory Cortex | 87.54% | S1 |
|  |  |  | 2 - Primary Somatosensory Cortex |  |  |
|  |  |  | 3 - Primary Somatosensory Cortex |  |  |
| CH03 | S2-D2 | 55/41/−8 | 46 - Dorsolateral prefrontal cortex | 26.25% | DLPFC |
| CH04 | S2-D7 | 60/22/5 | 46 - Dorsolateral prefrontal cortex | 36.22% | DLPFC |
| CH05 | S3-D2 | 42/62/−1 | 46 - Dorsolateral prefrontal cortex | 1.92% | DLPFC |
|  |  |  | 10 - Frontopolar area | 98.08% |  |
| CH06 | S3-D3 | 15/73/2 | 10 - Frontopolar area | 100.00% | MPFC |
| CH07 | S3-D8 | 31/64/22 | 10 - Frontopolar area | 100.00% | MPFC |
| CH08 | S4-D3 | −14/73/2 | 10 - Frontopolar area | 100.00% | MPFC |
| CH09 | S4-D4 | −41/60/−1 | 46 - Dorsolateral prefrontal cortex | 1.13% | DLPFC |
|  |  |  | 10 - Frontopolar area | 98.87% |  |
| CH10 | S4-D9 | −28/64/20 | 10 - Frontopolar area | 98.09% | MPFC |
| CH11 | S5-D4 | −53/39/−8 | 46 - Dorsolateral prefrontal cortex | 73.93% | DLPFC |
| CH12 | S5-D10 | −57/22/2 | 9 - Dorsolateral prefrontal cortex | 64.38% | DLPFC |
|  |  |  | 46 - Dorsolateral prefrontal cortex |  |  |
| CH13 | S6-D5 | −63/−43/46 | 1 - Primary Somatosensory Cortex | 91.14% | S1 |
|  |  |  | 2 - Primary Somatosensory Cortex |  |  |
|  |  |  | 3 - Primary Somatosensory Cortex |  |  |
| CH14 | S6-D11 | −51/−42/58 | 1 - Primary Somatosensory Cortex | 70.88% | S1 |
|  |  |  | 2 - Primary Somatosensory Cortex |  |  |
|  |  |  | 3 - Primary Somatosensory Cortex |  |  |
| CH15 | S7-D1 | 55/−18/60 | 6 - Pre-Motor and Supplementary Motor Cortex | 100.00% | M1 |
| CH16 | S7-D6 | 55/−40/58 | 1 - Primary Somatosensory Cortex | 54.24% | S1 |
|  |  |  | 2 - Primary Somatosensory Cortex |  |  |
|  |  |  | 3 - Primary Somatosensory Cortex |  |  |
| CH17 | S7-D12 | 41/−41/68 | 3 - Primary Somatosensory Cortex | 9.97% | S1 |
| CH18 | S7-D13 | 44/-20/68 | 6 - Pre-Motor and Supplementary Motor Cortex | 100.00% | M1 |
| CH19 | S8-D2 | 52/45/14 | 46 - Dorsolateral prefrontal cortex | 49.42% | DLPFC |
| CH20 | S8-D7 | 58/28/25 | 9 - Dorsolateral prefrontal cortex | 100.00% | DLPFC |
|  |  |  | 46 - Dorsolateral prefrontal cortex |  |  |
| CH21 | S8-D8 | 41/47/34 | 9 - Dorsolateral prefrontal cortex | 51.39% | DLPFC |
|  |  |  | 46 - Dorsolateral prefrontal cortex |  |  |
| CH22 | S9-D3 | 1/65/23 | 10 - Frontopolar area | 100.00% | MPFC |
| CH23 | S9-D8 | 16/58/39 | 10 - Frontopolar area | 37.65% | MPFC |
| CH24 | S9-D9 | −12/59/39 | 10 - Frontopolar area | 33.08% | MPFC |
| CH25 | S10-D4 | −49/46/12 | 9 - Dorsolateral prefrontal cortex | 39.67% | DLPFC |
|  |  |  | 46 - Dorsolateral prefrontal cortex |  |  |
| CH26 | S10-D9 | −38/48/32 | 9 - Dorsolateral prefrontal cortex | 81.82% | DLPFC |
| CH27 | S10-D10 | −56/28/23 | 9 - Dorsolateral prefrontal cortex | 93.08% | DLPFC |
|  |  |  | 46 - Dorsolateral prefrontal cortex |  |  |
| CH28 | S11-D5 | −63/−19/45 | 4 - Primary Motor Cortex | 9.50% | M1 |
|  |  |  | 6 - Pre-Motor and Supplementary Motor Cortex | 85.95% |  |
| CH29 | S11-D11 | −52/−20/61 | 6 - Pre-Motor and Supplementary Motor Cortex | 100.00% | M1 |
| CH30 | S12-D12 | 29/−43/74 | 3 - Primary Somatosensory Cortex | 8.49% | S1 |
| CH31 | S12-D13 | 32/−20/74 | 6 - Pre-Motor and Supplementary Motor Cortex | 100.00% | M1 |
| CH32 | S13-D11 | −38/−43/69 | 3 - Primary Somatosensory Cortex | 10.42% | S1 |
| CH33 | S13-D14 | −27/−44/74 | 3 - Primary Somatosensory Cortex | 1.96% | S1 |
| CH34 | S14-D11 | −41/−21/69 | 6 - Pre-Motor and Supplementary Motor Cortex | 100.00% | M1 |
| CH35 | S14-D14 | −30/−20/74 | 6 - Pre-Motor and Supplementary Motor Cortex | 100.00% | M1 |

Abbreviations: CH, channel; S, source; D, detector; M1, primary motor cortex; S1, primary somatosensory cortex; DLPFC, dorsolateral prefrontal cortex; MPFC, middle prefrontal cortex.

Special notes, the distance of each source and detector is 3cm and the MNI coordinates we listed is the midpoint of the channel. Therefore the overlap of brain regions is not absolute, but only probable. We divided the ROI by aligning the absolute overlap and functionally similar brain regions according to the SD layout
